# Update and recommendations on genetic testing for amyotrophic lateral sclerosis in clinical practice: a Brazilian expert view

**DOI:** 10.3389/fneur.2026.1928302

**Published:** 2026-07-31

**Authors:** Paulo Sgobbi, Wladimir Bocca Vieira de Rezende Pinto, Daniel Delgado Seneor, Marco Orsini, Marco Antônio Troccoli Chieia, Acary Souza Bulle Oliveira

**Affiliations:** 1 Motor Neuron Disease Unit, Division of Neuromuscular Diseases, Department of Neurology and Neurosurgery, Federal University of São Paulo (UNIFESP), São Paulo, SP, Brazil; 2 PSEG Centro de Pesquisa Clínica, São Paulo, SP, Brazil; 3 Faculty of Medicine, Iguaçu University, Nova Iguaçu, RJ, Brazil

**Keywords:** amyotrophic lateral sclerosis, genetic testing, genetics, motor neuron disease, next-generation sequencing, whole exome sequencing

## Abstract

Amyotrophic lateral sclerosis (ALS) is a complex and progressive neurodegenerative disorder characterized by the degeneration of both upper and lower motor neurons. Although most ALS cases occur sporadically, without a known family history of the disease, genetic factors play a major role in its pathogenesis through monogenic, oligogenic, or polygenic mechanisms. It is estimated that 10–15% of ALS cases occur in a familial setting; however, a specific monogenic cause cannot always be identified. Establishing the underlying genetic basis in both sporadic and familial ALS is essential, as it enables individualized and family genetic counseling, facilitates the early identification of at-risk or oligosymptomatic relatives, improves the prediction of gene-specific clinical trajectories, and, more recently, determines eligibility for gene-targeted therapies, such as tofersen for *SOD1*-associated ALS and ulefnersen, currently under clinical investigation, for *FUS*-associated ALS. Over the years, differing opinions have existed regarding the role of genetic testing in individuals diagnosed with ALS. However, accumulating clinical evidence has increasingly supported the timely and early implementation of genetic testing as part of the standard clinical management of patients with ALS. In this article, we present the perspective of leading Brazilian neurologists specializing in ALS care regarding the current role of genetic testing in clinical practice.

## 1 Introduction

Amyotrophic lateral sclerosis (ALS) is a fatal, progressive, and heterogeneous neurodegenerative disorder characterized by degeneration of upper and lower motor neurons, leading to motor and non-motor manifestations, progressive muscle weakness, and death from respiratory failure within 2–5 years after symptom onset. It is the most common adult-onset Motor Neuron Disease (MND). Although motor dysfunction predominates, up to 50% of patients develop cognitive and/or behavioral impairment, and approximately 15% develop frontotemporal dementia (FTD) (1–4).

ALS incidence increases with age, peaking between 60 and 79 years. Although improvements in diagnosis and reporting may influence incidence estimates, disease prevalence is expected to rise owing to population aging and advances in clinical management. Globally, the age-standardized incidence is 1.68 per 100,000 person-years, the lifetime risk is approximately 1 in 350, and an estimated 400,000 individuals will be living with ALS by 2040 (1–3, 5).

ALS results from complex interactions among aging, sex, environmental exposures, and genetic factors, with important implications for disease mechanisms, clinical research, and precision medicine (6). This article summarizes the genetic basis of ALS and current approaches to genetic testing, highlighting their role in improving diagnosis, prognosis, and personalized treatment.

## 2 Genetics of ALS

A glossary of key genetic terms related to ALS is provided in Table 1 (7–11). The first causative ALS gene was identified in 1993 through linkage and sequence analyses, which mapped a disease-associated locus on chromosome 21q and subsequently identified pathogenic variants in the copper-zinc superoxide dismutase 1 (*SOD1*) gene (7, 12, 13). *SOD1* remains one of the major genetic causes of ALS, accounting for up to 7% of sporadic cases and approximately 20% of familial cases with autosomal dominant or, less frequently, autosomal recessive inheritance (7, 12, 13).

**Table 1:** Summary of the main concepts and definitions related to genetics of ALS (7–11).

Concept	Definition and main characteristics
Familial ALS (5–15% of ALS cases)	Familial ALS may be classified according to Byrne criteria in three main categories: (i) Definite familial ALS: patient with two first-degree or second-degree relatives with ALS, or patient with one or more relative with ALS and a specific monogenic basis (gene variant) identified in the family; (ii) Probable familial ALS: patient with one first-degree or second-degree relative with ALS; (iii) Possible familial ALS: patient without a monogenic basis and no family history of ALS, or patient with one or more family members with FTD, or patient with a distant relative with ALS (e.g., great-aunts, second cousins). The most important genes include *C9orf72*, *SOD1*, *TARDBP*, and *FUS*. In Brazil, *VAPB* variants (mainly the p. Pro56Ser) represent a key mechanism associated with familial ALS. Some of the main *SOD1* variants include p. Ala5Val, p. Asp91Ala, p. Gly94Ala, p. Leu145Phe, p. Ile114Thr, p. Glu101Gly, and p. His47Arg. Some of the main *TARDBP* variants include p. Gly348Cys, p. Ala315Thr, p. Ala382Thr, p. Met337Val, p. Gly298Ser, p. Gly294Val, and p. Ile383Val. Some of the main *FUS* variants include p. Arg521Cys, p. Arg521His, p. Arg521Gly, p. Arg495Ter, p. Arg514Ser, and p. Arg522Gly.
Juvenile ALS	ALS case with motor symptoms starting before 25 years old. It represents a complex group with a genetically heterogeneous basis. Only 40% of juvenile ALS cases have a specific monogenic basis identified. The most important genes associated with juvenile ALS include *FUS*, *ALS2*, *SETX*, *SPG11*, *SIGMAR1*, *SPTLC1*, *SOD1*, and *UBQLN2*. Cases are more commonly sporadic. The most typical and aggressive *FUS* variant in juvenile ALS is p. Pro525Leu. Some of the main *SETX* variants include p. Thr3Ile and p. Leu389Ser. Some of the main *SIGMAR1* variants include p. Leu95fs* and p. Glu102Gln. Some of the main *SOD1* variants associated with juvenile ALS include p. Asn87Ser and p. Asp91Ala.
Young-onset ALS	ALS case with motor symptoms starting before 45 years old. Some of the most important genes associated with young-onset ALS include *SPG11*, *ALS2*, *SETX*, *SOD1*, and *FUS*. Both sporadic and familial ALS may be observed.

The major genetic forms of familial ALS and FTD-ALS are summarized in Table 2 (7, 9, 11, 14–17). ALS-associated genetic variants disrupt multiple cellular pathways involved in neurodegeneration, including glutamate excitotoxicity, impaired autophagy, protein misfolding and aggregation, mitochondrial dysfunction, oxidative stress, endosomal and vesicular trafficking defects, ubiquitin-proteasome system dysfunction, neuroinflammation, impaired axonal transport, and cytoskeletal abnormalities (2, 7, 9).

**Table 2:** Clinical, genetic and neuroimaging aspects related to familial ALS and FTD-ALS (7, 9, 11, 14–17).

Clinical subtype (# MIM number)	Pattern of inheritance; gene (locus)	Clinical, epidemiological, genetic, and neuroimaging characteristics
ALS type 1 (#105400)	AD / AR; *SOD1* (21q22.11)	Highly variable clinical course (rapidly or slowly progressive contexts). Mainly with typical ALS, rarely with juvenile ALS. Most commonly with LMN-dominant phenotypes. Cognitive disturbances are uncommon. Allelic to Progressive Spastic Tetraplegia and Axial Hypotonia (STAHP).
ALS type 2 (#205100)	AR; *ALS2* (2q33.1)	Mainly juvenile ALS. Most cases with UMN-dominant phenotypes, presenting with progressive spasticity, dysarthria, facial spasticity, pseudobulbar affect, and rarely with focal or generalized dystonia. Allelic to Infantile-onset Ascending Hereditary Spastic Paralysis (IAHSP).
ALS type 4 (#602433)	AD; *SETX* (9q34.13)	Adult-onset and juvenile ALS. Amyotrophy and distal-dominant weakness with pyramidal dysfunction signs. Clinical presentation resembling features of distal hereditary motor neuronopathy. Allelic to Ataxia-oculomotor apraxia type 2.
ALS type 5 (#602099)	AR; *SPG11* (15q21.1)	Juvenile ALS. Spastic paraparesis with brisk tendon reflexes, bulbar dysfunction, distal amyotrophy and weakness. Cognitive decline and behavioral changes may be observed. Neuroimaging findings: diffuse cortical atrophy, thin corpus callosum, and white matter involvement. Allelic to Hereditary Spastic Paraplegia type 11 and Charcot–Marie–Tooth disease type 2X.
ALS type 6 (#608030)	AD; *FUS* (16p11.2)	Variable age at onset: typical, juvenile and early-onset cases. Progressive clinical course, especially in juvenile and early-onset cases. Juvenile ALS associated with LMN-dominant phenotypes. Rare association with movement disorders in juvenile cases with the P525L variant. Typical cases may present with ALS, FTD-ALS, or rarely with long-standing ALS. Allelic to hereditary essential tremor type 4.
ALS type 8 (#608627)	AD; *VAPB* (20q13.32)	Typical ALS and early-onset cases. Slowly progressive LMN-dominant ALS with distal tremor and poliminimyoclonus, and lately with possible bulbar dysfunction and autonomic disturbance. Founder effect in Brazilian population (c.166C > T, p. Pro56Ser). Allelic to Finkel type non-5q Spinal Muscular Atrophy.
ALS type 9 (#611895)	AD; *ANG* (14q11.2)	Typical ALS, lately during course may be associated with FTD and parkinsonism.
ALS type 10 (#612069)	AD; *TARDBP* (1p36.22)	Typical ALS presentation, rarely with juvenile ALS. Spastic quadriparesis with brisk tendon reflexes, global amyotrophy, bulbar involvement, and may be associated with FTD, parkinsonism, and autonomic dysfunction.
ALS type 11 (#612577)	AD; *Fig4* (6q21)	Typical ALS with UMN-dominant phenotype (similar to PLS presentation), bulbar involvement, and pseudobulbar affect. Allelic to Charcot–Marie–Tooth disease type 4 J, Yunis-Varon syndrome, and Bilateral Temporooccipital Polymicrogyria.
ALS type 12 (#613435)	AD / AR; *OPTN* (10p13)	Typical ALS with LMN-dominant phenotype, bulbar compromise, and rarely with FTD. Allelic to Primary open angle glaucoma.
ALS type 13 (#183090)	AD; *ATXN2* (12q24.12)	Typical ALS with LMN-dominant phenotype, lately with bulbar dysfunction. Variable involvement with cerebellar ataxia. Neuroimaging findings: possible cerebellar atrophy or olivopontocerebellar atrophy and variable cortical atrophy. Intermediate range of CAG trinucleotide expansion repeat. Allelic to Spinocerebellar ataxia type 2.
ALS type 14 FTD-ALS type 6 (#613954)	AD; *VCP* (9p13.3)	Typical ALS, FTD or FTD-ALS with bulbar involvement. Intrafamilial variability including one or more individuals with ALS, FTD, Paget disease of bone, inclusion body myopathy, and parkinsonism. Neuroimaging findings: variable cortical atrophy, frontotemporal cortical atrophy. Allelic to Charcot–Marie–Tooth disease type 2Y and Inclusion body myopathy with early-onset Paget disease of bone and frontotemporal dementia type 1.
ALS type 15 (#300857)	XLD; *UBQLN2* (Xp11.21)	Juvenile ALS in male patients and late adult-onset cases in female patients. Typical ALS, which may be associated with FTD, chorea, athetosis, and dystonia. Neuroimaging findings: frontotemporal cortical atrophy.
ALS type 16 (#614373)	AR; *SIGMAR1* (9p13.3)	Juvenile ALS with slowly progressive spastic quadriparesis with brisk tendon reflexes and distal amyotrophy. Allelic to autosomal recessive distal hereditary motor neuronopathy type 2.
ALS type 17 FTD-ALS type 7 (#600795)	AD; *CHMP2B* (3p11.2)	Typical ALS with LMN-dominant phenotype, FTD, and FTD-ALS. May be associated with dystonia, myoclonus, and autonomic disturbances. Neuroimaging findings: variable frontoparietal cortical atrophy, variable white matter involvement.
ALS type 18 (#614808)	AD; *PFN1* (17p13.2)	Typical ALS with more prominent spinal-onset of symptoms. Allelic to Paget disease of bone type 7.
ALS type 19 (#615515)	AD; *ERBB4* (2q34)	Typical ALS. May be associated with parkinsonism.
ALS type 20 (#615426)	AD; *HNRNPA1* (12q13.13)	Typical ALS. Allelic to Distal myopathy type 3 and Inclusion body myopathy with early-onset Paget disease of bone without frontotemporal dementia.
ALS type 21 (#606070)	AD; *MATR3* (5q31.2)	Typical ALS with distal amyotrophy, bulbar compromise with vocal cord weakness, and possibly with FTD. Muscle biopsy may disclose rimmed vacuoles.
ALS type 22 FTD-ALS type 9 (#616208)	AD; *TUBA4A* (2q35)	Typical ALS. May be associated with FTD. Allelic to Autosomal dominant Spastic-ataxia type 11 and Congenital myopathy type 26.
ALS type 23 (#617839)	AD; *ANXA11* (10q22.3)	Typical ALS in the elderly, generally with no significant cognitive involvement or FTD. Incomplete penetrance. Allelic to Inclusion body myopathy and brain white matter abnormalities.
ALS type 24 (#617892)	AD; *NEK1* (4q33)	Typical ALS with LMN-dominant compromise in the elderly and adult groups. Bulbar involvement. Neuroimaging findings: Variable cortical atrophy, temporal lobe atrophy. Allelic to Orofaciodigital syndrome type II and Short-rib thoracic dysplasia type 6.
ALS type 25 (#617921)	AD; *KIF5A* (12q13.3)	Typical ALS with slowly progressive course. Incomplete penetrance. Allelic to Hereditary Spastic Paraplegia type 10 and Intractable neonatal myoclonus.
ALS type 26 (#619133)	AD; *TIA1* (2p13.3)	Typical ALS. May be associated with FTD and severe bulbar involvement. Amnestic involvement may be observed. Incomplete penetrance. Allelic to Welander distal myopathy.
ALS type 27 (#620285)	AD; *SPTLC1* (9q22.31)	Juvenile ALS with bulbar dysfunction, feet and hammer toe deformity, and executive dysfunction. Allelic to Hereditary sensory and autonomic neuropathy type IA.
ALS type 28 (#620452)	AD; *LRP12* (8q22.3)	Typical ALS with slowly progressive phenotype. Associated with 60–100 (CGG) trinucleotide repeat expansion. Allelic to Oculopharyngodistal myopathy type 1.
FTD-ALS type 1 (#105550)	AD; *C9orf72* (9p21.2)	Typical ALS, FTD, or FTD-ALS, generally with rapidly progressive phenotype. May be associated with supranuclear gaze palsy, Huntington disease-like phenotype, and parkinsonism. Neuroimaging findings: frontotemporal cortical atrophy, thalamic atrophy. Pathological (GGGGCC) hexanucleotide repeat expansion.
FTD-ALS type 2 (#615911)	AD; *CHCHD10* (22q11.23)	Typical ALS. May be associated with FTD, parkinsonism, eyelid ptosis, cerebellar ataxia, and sensorineural deafness. Neuroimaging findings: variable cortical atrophy; frontotemporal atrophy. Muscle biopsy: chronic neurogenic amyotrophy, subsarcolemmal mitochondrial proliferation. Allelic to Jokela type non-5q Spinal Muscular Atrophy and Isolated mitochondrial myopathy.
FTD-ALS type 3 (#616437)	AD; *SQSTM1* (5q35.3)	Typical ALS, FTD, or FTD-ALS. May be associated with Paget disease of bone and apraxia. Neuroimaging: diffuse cortical atrophy. Allelic to Distal myopathy with rimmed vacuoles, Paget disease of bone type 3, and Neurodegeneration with ataxia, dystonia, and gaze palsy.
FTD-ALS type 4 (#616439)	AD; *TBK1* (12q14.2)	Typical ALS, FTD, or FTD-ALS. Bulbar involvement. Neuroimaging findings: cortical atrophy. Allelic to Autoinflammation with arthritis and vasculitis.
FTD-ALS type 5 (#619141)	AD; *CCNF / FBXO1* (16p13.3)	Typical ALS, FTD, or FTD-ALS. Age-dependent penetrance.
FTD-ALS type 8 (#619132)	AD; *CYLD* (16q12.1)	Typical ALS, FTD, or FTD-ALS. Amnestic involvement may be also present. Neuroimaging findings: frontotemporal cortical atrophy. Allelic to Brooke-Spiegler syndrome, Multiple familial trichoepithelioma type 1, and Familial Cylindromatosis.

AD, Autosomal dominant; ALS, Amyotrophic Lateral Sclerosis; AR, Autosomal recessive; FTD, Frontotemporal Dementia; IAHSP, To Infantile-onset ascending hereditary spastic paralysis; LMN, Lower motor neuron; MIM, Mendelian Inheritance in Man; PLS, Primary Lateral Sclerosis; STAHP, Progressive Spastic Tetraplegia and Axial Hypotonia; UMN, upper motor neuron; XLD, X-linked dominant.

There are substantial differences in the genetic architecture underlying sporadic and familial ALS across different populations. Globally, pathogenic expansions in *C9orf72* represent the most frequent genetic cause of both sporadic (5–10% of cases) and familial ALS (30–40% of cases), particularly among individuals of European ancestry, with the highest prevalence observed in Northern Europe, Scandinavia, and the United Kingdom. In contrast, *C9orf72* expansions are exceedingly rare in Asian populations (7, 13).

Pathogenic variants in *SOD1* have relatively high frequencies reported in the United States, several European countries (including Germany and Italy), and Brazil. Variants in *TARDBP* are responsible for approximately 3–5% of familial ALS and about 1% of sporadic ALS, occurring more frequently in Southern European populations, particularly in Italy, France, and other Mediterranean countries. *FUS* variants account for fewer than 5% of familial ALS cases and less than 1% of sporadic ALS cases but are comparatively more prevalent in East Asian populations, especially in China and Japan (7, 13).

In the Brazilian population, pathogenic variants in *VAPB*, *C9orf72*, and *SOD1* are among the most frequently identified genetic causes of ALS. The relatively high prevalence of *VAPB* variants is largely attributable to a well-established founder effect, whereas *C9orf72* expansions are predominantly observed in families of European ancestry. Additionally, pathogenic variants in *TBK1* and *NEK1* are more commonly reported in European and North American populations, while *OPTN* variants are disproportionately represented in Japanese and Chinese cohorts (7, 13).

## 3 Genetic methods in ALS

ALS genetic testing is not included as a core component of the current Gold Coast diagnostic criteria (18). However, the identification of pathogenic variants in well-established ALS genes, particularly during the early or presymptomatic stage, may facilitate differential diagnosis and diagnostic work-up (19, 20). More broadly, genetic testing enables a definitive molecular diagnosis, reduces unnecessary investigations, supports access to gene-specific therapies (e.g., tofersen for *SOD1*-associated ALS), facilitates enrollment in genotype-driven clinical trials, and improves prognostic assessment, surveillance strategies, and genetic counseling regarding familial recurrence (21, 22). Appropriate test selection and careful interpretation remain essential to avoid misclassification and inappropriate clinical management of patients and their relatives (21, 23, 24).

Genetic testing is highly valued by both familial and sporadic ALS patients and has substantially advanced the understanding of ALS pathophysiology (25, 26). Current recommendations are supported by studies evaluating different testing strategies as well as international guidelines, expert consensus statements, and systematic reviews (27, 28). Approximately 10–15% of ALS cases harbor pathogenic variants in a single causative gene (15).

NGS-based screening studies have also demonstrated the occurrence of oligogenic inheritance in ALS. In a Sheffield cohort predominantly comprising sporadic cases, pathogenic or likely pathogenic variants in two or more ALS-associated genes were identified in 13% of individuals (29). Similarly, a study from Washington University at St. Louis detected pathogenic variants in multiple ALS-related genes in 3.8% of patients (30). These findings have important implications for molecular diagnosis, genetic counseling, and risk assessment.

A multicenter German study involving 11 ALS referral centers (2021–2024) further demonstrated the clinical value of broad genetic testing (31). Among 1,935 screened patients, nearly half opted to receive treatment-relevant genetic information, enabling identification of *SOD1* pathogenic variants and access to expanded-use tofersen therapy. Notably, 68% of *SOD1* carriers had no family history of ALS, and the median interval from genetic testing to treatment initiation was 94 days (31).

Genetic testing should therefore be performed as early as possible after ALS diagnosis. In one specialized London center, 14% of patients died before receiving their genetic test results, including one patient carrying an actionable *SOD1* pathogenic variant (32).

### 3.1 Next,-generation sequencing-based gene panels

NGS-based multigene panel testing for ALS has completely transformed the history about the genetic and pathophysiological mechanisms in both research and clinical practice, especially in the last two decades, and has become a key diagnostic first-tier approach for clinicians (27). NGS technique uses a massive parallel processing approach which generates hundreds of megabases of sequence reads, after initial clonal in-situ amplification and sequencing by DNA synthesis (33). Generally, NGS-based gene panels should always seek to include the main emblematic genes associated with both sporadic and familial forms of pathogenic variants and that are related to the pathophysiological basis of the disease under study (21). Also, the indication of the best coverage of genes included in gene panels when investigating sporadic or familial cases of ALS must always consider genetic aspects related to the epidemiological profile in each population (17, 34, 35). In the context of familial and sporadic ALS, it is very common to use NGS-based panels that include genes related to diseases with a pathophysiological basis overlapping with ALS, such as Hereditary Spastic Paraplegia, FTD and eventually genes associated with some forms of genetic parkinsonism and inherited neuropathies. Gene panels usually tend to have a much smaller and less significant number of incidental findings, variants of uncertain significance (VUS) and secondary findings than Whole-exome sequencing. It should be noted that it is essential in clinical practice to verify the genes that are part of the analysis of different NGS-based panels available (21).

A previous genetic screening study in a group of patients with ALS disclosed significant genetic results in almost 21% of cases, in 15% of sporadic cases, and also 21% of cases showed additional VUS in ALS-related genes (29). It is of notice that, in a study conducted at the Ghent University Hospital, the use of exome sequencing-based multigene panels (and not the traditional pure NGS-based panel) gave rise to low rates of definite conclusive identifiable variants in ALS and more than 20% of VUS identified in ALS patients, disclosing, thus, the potential limitation in the routine use of this approach derived from exome sequencing data (36). Considering only the familial ALS group of patients, NGS-based multigene panels identified significant pathogenic variants in 45.5% of cases (37).

Despite the good diagnostic profile performed by multigene panels, it is important to recognize their potential limitation in identifying large duplications or deletions, epigenetic changes, mosaicism, repeat expansion disorders and repeat contraction diseases, although these generally represent scenarios of minor importance in the context of sporadic and familial ALS, except for the representation of the *C9orf72* gene. In rare situations where large deletions and duplications are highly suspected or in NGS-based panels with low sensitivity to detect copy number variations (CNVs), multiplex ligation-dependent probe amplification (MLPA) or cytogenetic genomic hybridization (CGH) arrays may be considered by the clinician (21).

### 3.2 Genetic analysis of C9orf72 hexanucleotide repeat expansion

*C9orf72* assay is frequently used concurrently to the gene panel evaluation during the first steps of genetic testing both in sporadic and familial ALS (27). Pathological hexanucleotide repeat expansions may be detected by the analysis of *C9orf72* gene using of polymerase chain reaction (PCR) and capillary electrophoresis followed by repeat-primed PCR assay. Other techniques, such as Southern blot, expansion Hunter analysis of PCR-free whole-genome sequencing (WGS) data, and dual-mode PCR, may be also used to identify expanded alleles (27, 38). *C9orf72* pathologic hexanucleotide repeat expansions account for around 33.7% of European familial ALS cases, 5.1% of European sporadic ALS cases, 2.3% of Asian familial ALS cases, and 0.3% of Asian sporadic ALS cases (16, 39). According to previous study with a Brazilian cohort, it is estimated a representation of 22% of familial ALS cases due to *C9orf72* hexanucleotide repeat expansions (40).

### 3.3 Single gene testing and sanger sequencing

Single gene testing using Sanger method sequencing is currently supported for use in cases in which there is a high-suspicion index for a specific monogenic basis associated with ALS, such as in cases with a highly suspected genetic basis due to familial phenotypes or epidemiological contexts. This is associated with high-diagnostic yield, for example, in Brazilian patients with long-standing lower motor neuron-dominant ALS and an autosomal dominant family history, suggesting familial ALS type 8 and the need for *VAPB* gene sequencing (7, 11). Sanger sequencing enables the proper evaluation of short segments and the detection of small indels and point mutations, however important limitation exists for the identification of large deletions, nucleotide repeat expansion and structural variants such as copy number variation (33). Another important context is the role of Sanger sequencing to confirm and validate variant results obtained in NGS-based testing. Sanger sequencing method, however, has several concerns and limitations that makes its indication more restricted nowadays, including the low rate of detection (especially in sporadic cases), the limitation to perform massive parallel sequencing approaches, the low sensitivity (especially due to the very small targeted regions evaluated in the genome), the limitation to identify large duplications or deletions, and the worse cost effectiveness, if compared to NGS-based approaches.

### 3.4 Common variant testing

Common variant testing is currently rarely used in clinical practice, but may be used in scenarios of high suspicion of genetic subtypes related to genetic variants with high incidence in specific population contexts or even founder effects, such as the c.166C > T (p. Pro56Ser) variant in heterozygosity in the *VAPB* gene (NM_004738.5) in the population of southeastern Brazil in the Zona da Mata region (41).

### 3.5 Targeted variant testing

Similarly, targeted variant testing may be used in family members of patients with a definite genetic basis associated with a well-defined pathogenic variant. Targeted variant testing for pathogenic variants is generally faster and less costly than other genetic testing methods and technically less complicated than NGS-based panels and exome and genome sequencing. Targeted variant analysis does not enable proper identification of additional genetic risk factors or concomitant pathogenic variants in other genes related to ALS, which may synergistically participate in the pathogenesis of individual cases (42).

### 3.6 Whole-exome sequencing

Whole-exome sequencing (WES) for ALS is commonly used in clinical practice. Clinical exome sequencing has also become one of the standards of care genetic methods during the diagnostic work-up of neurogenetic disorders (43). Previous studies performed WES in many patients with ALS, enabling the identification of novel genes and variants associated with ALS as well as genes related to risk factors and the putative role in its pathogenesis (44). A study performed in the Oxford MND Clinic offering *C9orf72* expansion analysis followed by WES in patients with ALS and PLS disclosed 11.3% of pathogenic variants identified in *C9orf72*, *SOD1*, *TARDBP*, *FUS* and *TBK1* genes (45). Furthermore, WES enabled the identification of high-risk variants for ALS and common missense variants and polymorphisms linked to ALS risk (45). The routine use of these methods to identify both genetic risk factors, modifiers and protective genetic variants is not the main objective of this sequencing technology, however several distinct pathways and risk genes have been identified by WES (44, 46, 47). WES may be of special interest in cases with atypical or multisystemic presentations, in contexts in which there are no identifiable genotype–phenotype correlations or in which there are no previously identified monogenic basis associated with a group of genes available for evaluation in NGS-based panels (21).

### 3.7 Whole-genome sequencing

WGS could represent a potential alternative in the diagnostic work-up of specific cases in which large and complex structural variants or deep intronic variants should be considered, however these scenarios represent a very small proportion of ALS cases. Given the high costs associated with WGS, the complex variants arising from sequencing, the interpretation profile uncertain for the high percentage of variants identified (21) and especially the existence of a polygenic contribution in ALS pathogenesis that is still undefined regarding its importance, WGS has not been routinely used in ALS diagnostic work-up, especially in sporadic cases. A multicenter study involving ALS patients in Italy disclosed 43.9% of identifiable variants in early-onset cases and 19.7% in late-onset cases as well as 14.6% of cases with any type of worse prognosis genetic factors (48).

### 3.8 Miscellaneous – other genes and methods used

Other complex genetic mechanisms linked to *RFC1*, *ATXN2*, *LRP12* and other nuclear genes have been implicated in ALS. Abnormal and complex pentanucleotide repeat expansion in *RFC1* (4p14) has been linked to Cerebellar Ataxia, Neuropathy, and Vestibular Areflexia syndrome (CANVAS), however in other reports expansions have been associated with motor neuron involvement and sensory neuropathy (49). Motor neuron involvement in neuropathological changes has been also associated with abnormal expansions seen in CANVAS (50). Intermediate CAG trinucleotide repeat expansion in the *ATXN2* gene (12q24.12) ranging from 27 to 31 repeats has been associated with ALS (51). Abnormal trinucleotide repeats expansion (CGG) in the *LRP12* (8q22.3) gene has been associated with autosomal dominant ALS type 28 in sporadic and familial contexts in Japan (52).

## 4 Discussion and practical recommendations

Current guidelines addressing genetic testing in ALS remain limited (27, 53, 54). Genetic evaluation should be individualized and integrated with clinical, neuroimaging, and neurophysiological findings to guide test selection and variant interpretation. Clinicians should also consider the benefits and limitations of testing, including gene penetrance, psychosocial implications, and the impact of results on patients and their families (55).

**Figure 1:**
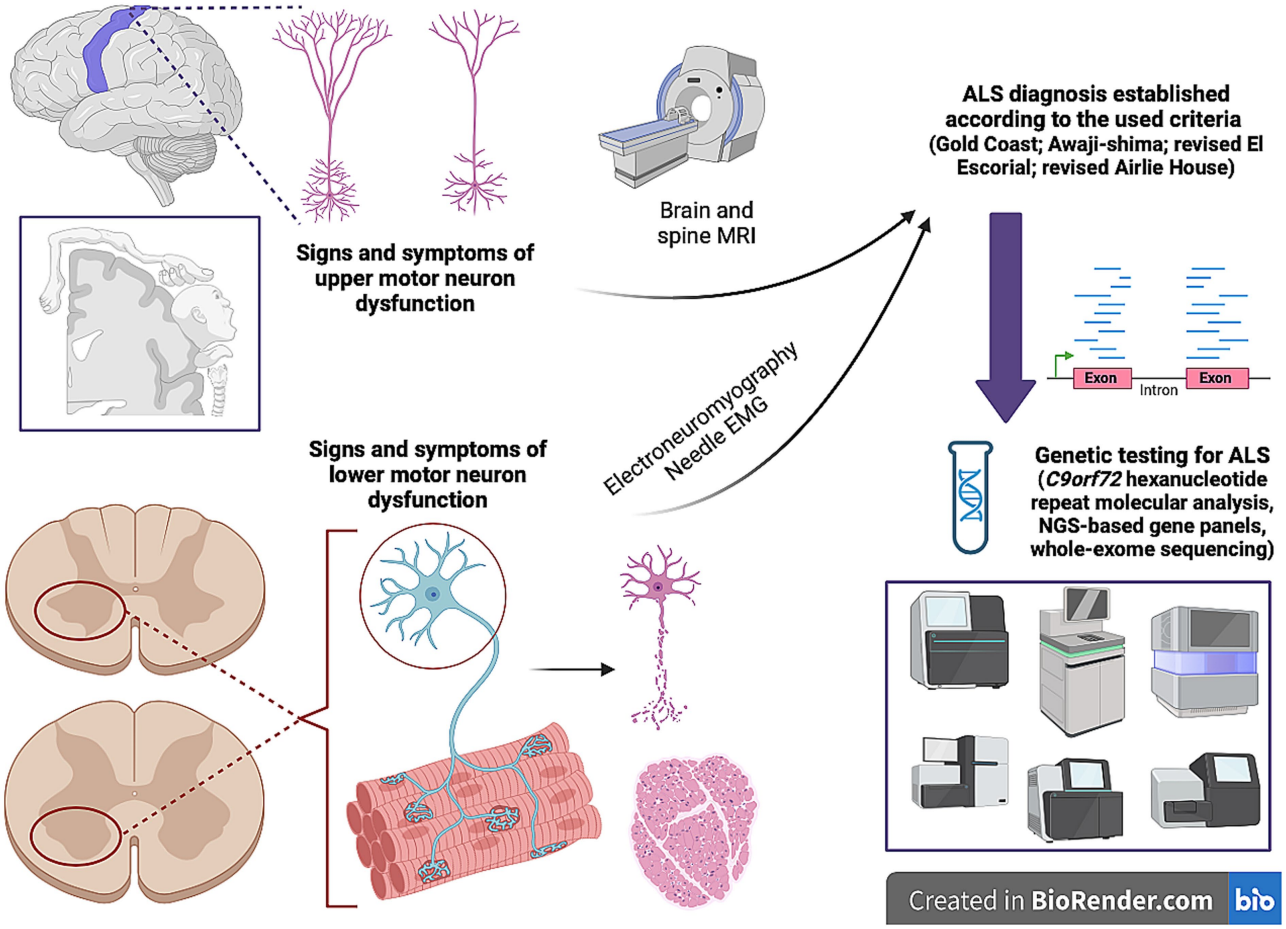
Genetic testing for symptomatic patients with sporadic and familial ALS. Genetic testing does not rule out the need of initial clinical evaluation looking up for signs and symptoms associated with upper and lower motor neuron dysfunction as well as the basic diagnostic work-up. NGS, Next-generation sequencing (Created with BioRender.com).

The main set of recommendations is presented as follows:

*Practical Recommendation 1*. Genetic testing should be offered for all patients with familial or sporadic ALS (Figure 1), in accordance with the international ALS Genetic Testing and Counseling Guidelines Expert Panel (27) and recent expert opinion-based reviews (28) and recommendations (15, 28, 45).
*Practical Recommendation 2*. Initial genetic evaluation should include testing for *C9orf72* hexanucleotide repeat expansion and at least the *SOD1*, *FUS*, and *TARDBP* genes (27).
*Practical Recommendation 3*. NGS-based multigene panels or WES should be considered in familial ALS without an established monogenic cause. Although no international consensus exists regarding the optimal gene panel composition, it should include the major ALS-associated genes according to current evidence and local epidemiological characteristics (24, 28, 35, 56).
*Practical Recommendation 4*. Targeted variant testing is recommended for symptomatic relatives of patients carrying a known pathogenic variant.
*Practical Recommendation 5*. Genetic testing strategies should consider population-specific epidemiological characteristics. For example, *VAPB* gene testing is recommended in Brazilian patients with slowly progressive autosomal dominant familial ALS (7).
*Practical Recommendation 6*. Gene selection for NGS panels should be guided by phenotype, inheritance pattern, multisystem involvement (e.g., inclusion body myopathy, Paget disease of bone), associated neurological manifestations (e.g., FTD, Parkinsonism, cerebellar ataxia), and population-specific epidemiology (7, 11). When there is a family history of FTD, dementia, Parkinsonism, or related neurodegenerative disorders, genes with clinical and pathogenic overlap should also be included (57).
*Practical Recommendation 7*. Pre- and post-test genetic counseling is recommended. Variant interpretation should follow the American College of Medical Genetics and Genomics and the Association for Molecular Pathology criteria (58), integrating genotype, phenotype, and family history (59). VUS should be interpreted cautiously and periodically reassessed (33, 60, 61).
*Practical Recommendation 8*. Presymptomatic genetic testing may be considered on an individual basis for high-risk relatives after careful assessment of benefits and limitations (19, 20). Furthermore, it may also be appropriate for enrollment in gene-specific clinical trials, such as the Phase 3 ATLAS trial for presymptomatic *SOD1* variant carriers (62). Individuals carrying pathogenic variants should undergo regular clinical surveillance and receive lifestyle counseling (63). Testing asymptomatic relatives for VUS or variants with incomplete penetrance requires particular caution (15).
*Practical Recommendation 9*. Genetic testing should complement, but never replace, the clinical diagnosis of ALS. When ALS remains unconfirmed or alternative MND are suspected, additional genetic investigations should be guided by the differential diagnosis, including: (i) *SMN1* and *SMN2* genes analysis by MLPA for 5q Spinal Muscular Atrophy; (ii) CAG trinucleotide repeat expansion analysis in the *AR* gene for Kennedy’s disease; (iii) NGS panels or WES for non-5q Spinal Muscular Atrophy or Hereditary Spastic Paraplegia; (iv) repeat expansion analysis for hereditary ataxias (e.g., CAG repeats in *ATXN2* gene; pentanucleotide repeats in *RFC1* gene; GGCCTG hexanucleotide repeats in *NOP56* gene).
*Practical Recommendation 10*. Age at symptom onset should not be used as the sole criterion to exclude genetic testing (64), as juvenile and childhood-onset ALS may occur in both familial and sporadic cases (9).

## 5 Conclusion

Genetic testing has become a fundamental component of ALS care in both familial and sporadic disease. Testing should be individualized, accompanied by appropriate pre- and post-test genetic counseling, and incorporated into routine neurological practice. Broad genetic testing is recommended whenever feasible, with at least *C9orf72*, *SOD1*, *FUS*, and *TARDBP* included in the initial evaluation.

## Data Availability

The original contributions presented in the study are included in the article/supplementary material, further inquiries can be directed to the corresponding author.
